# Distinguishing epigenetic features of preneoplastic testis tissues adjacent to seminomas and nonseminomas

**DOI:** 10.18632/oncotarget.7074

**Published:** 2016-01-29

**Authors:** Ildar V. Gainetdinov, Sofia A. Kondratieva, Yulia V. Skvortsova, Marina V. Zinovyeva, Elena A. Stukacheva, Alexey Klimov, Alexey A. Tryakin, Tatyana L. Azhikina

**Affiliations:** ^1^ Department of Genetics and Postgenomic Technologies, Shemyakin-Ovchinnikov Institute of Bioorganic Chemistry, Russian Academy of Sciences, Moscow, Russia; ^2^ Department of Oncology, Blokhin Russian Cancer Research Center, Moscow, Russia; ^3^ Department of Clinical Pharmacology and Chemotherapy, Blokhin Russian Cancer Research Center, Moscow, Russia

**Keywords:** PIWI, testicular germ cell tumor, DNA methylation, LINE-1, DDX4

## Abstract

PIWI pathway proteins are expressed during spermatogenesis where they play a key role in germ cell development. Epigenetic loss of PIWI proteins expression was previously demonstrated in testicular germ cell tumors (TGCTs), implying their involvement in TGCT development. In this work, apart from studying only normal testis and TGCT samples, we also analyzed an intermediate stage, i.e. preneoplastic testis tissues adjacent to TGCTs. Importantly, in this study, we minimized the contribution of patient-to-patient heterogeneity by using matched preneoplastic/TGCT samples. Surprisingly, expression of germ cell marker *DDX4* suggests that spermatogenesis is retained in premalignant testis tissues adjacent to nonseminoma, but not those adjacent to seminoma. Moreover, this pattern is followed by expression of PIWI pathway genes, which impacts one of their functions: DNA methylation level over LINE-1 promoters is higher in preneoplastic testis tissues adjacent to nonseminomas than those adjacent to seminomas. This finding might imply distinct routes for development of the two types of TGCTs and could be used as a novel diagnostic marker, possibly, noninvasively. Finally, we studied the role of CpG island methylation in expression of PIWI genes in patient samples and using *in vitro* experiments in cell line models: a more complex interrelation between DNA methylation and expression of the corresponding genes was revealed.

## INTRODUCTION

Testicular germ cell tumors (TGCTs) are heterogeneous cancers classified into less invasive seminomas and more aggressive nonseminomas and are believed to be caused by both genetic and environmental factors [1]. Apart from distinct histological features, seminomas and nonseminomas also differ in their prognosis and choice of treatment strategy [2]. These two types of TGCTs were shown to have numerous epigenetic differences and distinguishing expression of various biomarkers [3, 4].

Essentially, all TGCTs are deemed to arise because of failure to undergo normal spermatogenesis and through an intermediate stage of carcinoma *in situ* [5–7]. One of the key players in spermatogenesis is PIWI pathway responsible for epigenetic silencing of retrotransposons [8, 9]. Importantly, PIWI proteins have been also implicated in development of various types of cancers [10, 11]. Recently, Ferreira *et al.* [12] demonstrated CpG island (CGI) hypermethylation and a concomitant decrease in expression level of PIWI pathway genes in TGCTs compared to the testes of healthy individuals, suggesting a role of PIWI in TGCT tumorigenesis.

In this work, we extended these data by studying an intermediate preneoplastic stage of TGCT development, which contains carcinoma *in situ*, i.e. premalignant testis tissues adjacent to TGCTs. We have found distinguishing features of spermatogenesis and PIWI machinery function in the two types of TGCTs. Firstly, expression of germ cell marker *DDX4* suggests that extent of spermatogenesis in premalignant testis adjacent to nonseminoma is comparable to that in healthy testis. On the other hand, premalignant testis adjacent to seminoma display downregulation of *DDX4* expression. Furthermore, expression of *PIWIL1/2/4* and DNA methylation level of LINE-1 promoters were found to coincide with this pattern: higher in tissues adjacent to nonseminoma than seminoma. This finding might imply different causes for development of these two types of TGCTs and could be used as a novel diagnostic marker. We additionally studied epigenetic regulation of PIWI machinery genes expression in patient samples and *in vitro* in cell line models: role for *PIWIL1/2* CGI methylation appears to be more complex than it was proposed.

## RESULTS AND DISCUSSION

### Expression levels of germ cell marker DDX4 and PIWI genes are different in preneoplastic testis tissues adjacent to seminoma and nonseminoma

**Figure 1:**
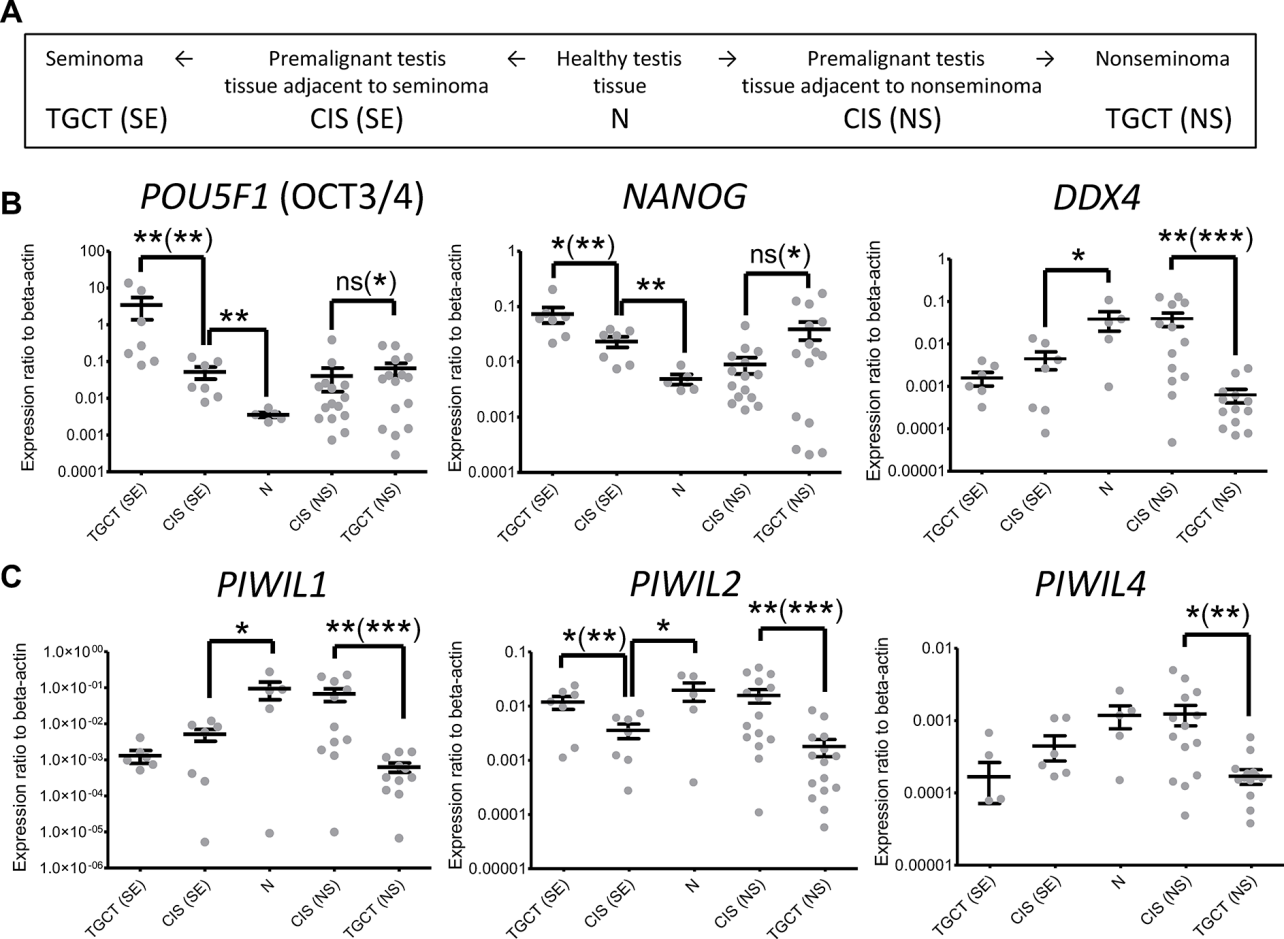
Expression of CIS and germ cell markers and PIWI protein genes in testis and testicular germ cell tumors (**A**) Schematic description of the sample types used in the work and the approach to study normal-to-malignant transition. (**B**) Expression of carcinoma *in situ* markers *POU5F1* (OCT3/4) and *NANOG* and germ cell marker *DDX4*. (**C**) Expression of PIWI protein genes *PIWIL1*, *PIWIL2*, and *PIWIL4*. Average value and SEM (Standard Error of the Mean) are shown for all samples. *P* value summary for Mann-Whitney non-paired *U* test and paired Wilcoxon signed-rank test (in brackets) are shown only for significant differences. Due to logariphmic scale several near-zero values are not depicted: *DDX4* – 1 TGCT(SE), *PIWIL1* – 1 TGCT(SE), *PIWIL4* – 3 TGCT(SE), 1 CIS (SE), 1 CIS (NS) and 2 TGCT(NS).

In order to gain more insights into the role of PIWI machinery in TGCT tumorigenesis, apart from just normal and TGCT samples we also used an intermediate preneoplastic stage - testis tissues adjacent to TGCT (CIS samples). This way, we tracked normal-to-malignant transition: from N samples (healthy testis tissues, *n* = 5) through CIS samples (carcinoma *in situ*-riddled testis tissues adjacent to TGCT, *n* = 22) to overt TGCT samples both for seminomas and for nonseminomas (Figure 1A).

Importantly, since each CIS sample was matched to the corresponding TGCT, we were also able to take into account patient-to-patient heterogeneity, which is an emerging feature of tumorigenesis [13–15]. Additionally, we looked into expression of germ cell marker *DDX4* (human homologue of Drosophila VASA) to assess for the extent of spermatogenesis and carcinoma *in situ* markers *POU5F1* (OCT3/4) and *NANOG* to evaluate the degree of testicular dysgenesis in the samples [16, 17]. This way we could confirm estimates about the histological composition of the samples under study.

Firstly, expression level of carcinoma *in situ* markers gradually increases from N through CIS samples to TGCT for both seminomas and nonseminomas (Figure 1B), which reflects accumulation of carcinoma *in situ* in tissues along the normal-to-malignant transition.

Secondly, although expression of *DDX4* is significantly lower in TGCT samples compared to normal testis tissues (Figure 1B) implying deteriorated spermatogenesis, this germ cell marker expression is mostly retained in preneoplastic testis tissues adjacent to nonseminoma unlike those adjacent to seminoma. Importantly, this pattern appears to be followed by PIWI proteins (Figure 1C). Spearman's correlation coefficient for the expression level of *PIWIL1/2/4* and *DDX4* genes in normal and premalignant tissues (N and NT) is as high as 0.85–0.99 (Supplementary Data File 1), suggesting that expression of PIWI proteins, in fact, reflects the content of germ cells in the samples. It might also imply coordinated regulation of *DDX4* and PIWI proteins expression, which is in agreement with the fact that DDX4 has been shown to be involved in piRNA pathway [18].

Thus, by comparing not only healthy testis tissues (N samples) and TGCTs but also through studying an intermediate premalignant stage represented by testis tissue adjacent to tumors (CIS samples) we revealed the distinguishing expression patterns for both *DDX4* and PIWI proteins. Specifically, these genes are still expressed in CIS samples adjacent to nonseminomas, but are downregulated in those adjacent to seminomas, which could be indicative of different tumorigenesis routes for these two types of TGCTs. Possibly, loss of PIWI machinery function is a prerequisite of germ cell malignization into seminoma, unlike nonseminoma.

One of the prominent features of the obtained data is remarkable patient-to-patient variability, even among normal testes (Ns, Figure 1B and 1C). This is also evident in the cohort in the study by Ferreira *et al.* [12]. In order to assess the degree of gene expression heterogeneity among patients, we also performed paired statistical testing (Wilcoxon signed-rank test) of matched CIS/TGCT samples (Figure 1B and 1C). The results give more significant evidence (lower *P* values) to make conclusions on patterns of expression changes. While the expression level of *PIWIL1/2/4*, *DDX4* and carcinoma *in situ* markers is highly variable among patients, the change patterns in CIS/TGCT matched samples are more consistent across the cohort if regarded in each patient individually. Therefore, the use of matched samples allows minimizing contribution of expression heterogeneity between patients.

We next investigated whether the expression of *PIWIL1, PIWIL2 or PIWIL4* in TGCTs correlated with that of *SOX2* or *SOX17*. These two SOX gene family members play important biological roles in germ cell development: *SOX2* is involved in maintaining pluripotency [19, 20], while *SOX17* is a key specification factor for the germ cell fate [21]. Their functions are also relevant to the histological features of TGCTs: more pluripotent embryonal carcinomas (nonseminomas) overexpress *SOX2* and more germ cell-like seminomas overexpress *SOX17*. Consequently, these proteins were previously proposed as markers for the two types of TGCTs [22].

Our analysis showed that the most significant correlation was between *PIWIL2* and *SOX17* (Supplementary Data File 2). This fact is consistent with a more germ cell-like gene expression pattern in seminomas [23], since *PIWIL2* is a component of piRNA pathway, whose expression is typically restricted to the germ line. Importantly, *PIWIL2* expression was upregulated in seminomas and downregulated in nonseminomas compared to the matched CIS samples (Figure 1C). Accordingly, *PIWIL2* transcription level was higher in seminomas than in nonseminomas (Supplementary Data File 3) as in previous reports [24, 25]. This *PIWIL2* expression pattern was also observed in The Cancer Genome Atlas cohort (Supplementary Data File 3). This finding might have some biological relevance, since PIWIL2 was shown to be involved in carcinogenesis as well [10, 11, 26].

**Figure 2:**
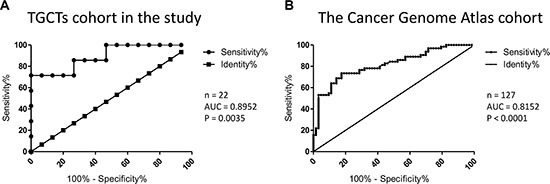
Assessment of sensitivity and specificity of *PIWIL2* expression level as a biomarker to distinguish between seminomas and nonseminomas ROC curve analysis was performed and AUC (Area Under the Curve) was calculated for the cohorts from the present study (**A**) and The Cancer Genome Atlas project (**B**). The number of samples in each cohort and *P* values are indicated.

We performed ROC curve analysis in order to assess sensitivity and specificity of *PIWIL2* expression level as a biomarker to distinguish between seminomas and nonseminomas. AUC (area under the curve) values for the cohort used in this study (Figure 2A) and for The Cancer Genome Atlas cohort (Figure 2B) indicate that *PIWIL2* could be a possible candidate to extend current panels of routinely used TGCT biomarkers [4].

### LINE-1 promoter methylation level is affected by PIWI genes expression and could serve as a novel diagnostic marker

**Figure 3:**
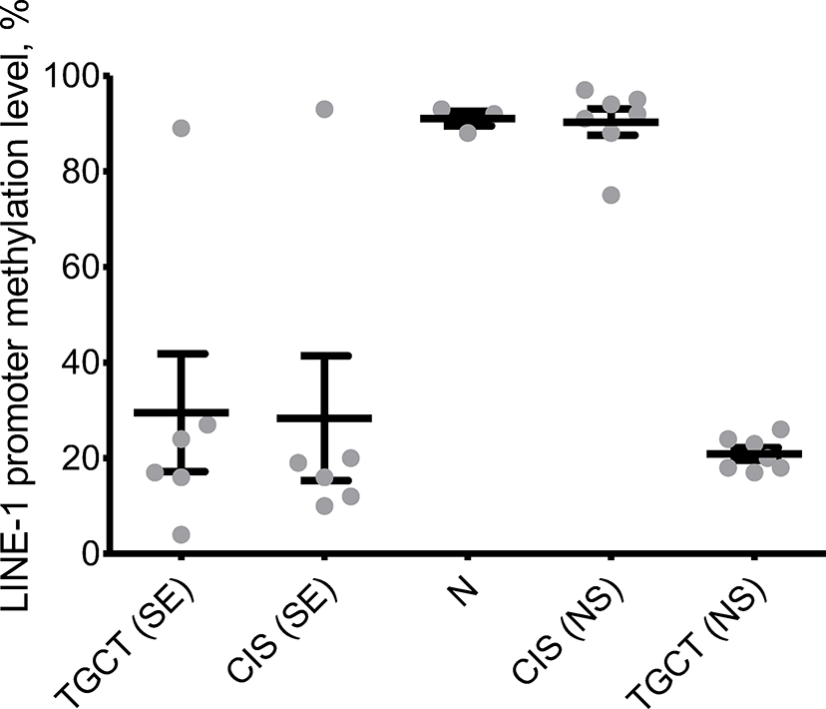
Integral methylation status of LINE-1 CpG islands Melting curve analysis was used to obtain methylation level in healthy testis tissues (N), premalignant testis tissues (CIS samples) and testicular germ cell tumors (TGCT). SE – seminoma, NS – nonseminoma.

One of the major roles of PIWI proteins in spermatogenesis is maintenance of genomic stability through epigenetic silencing of retrotransposons [27]. It is known that deregulation of retrotransposon expression may lead to neoplastic transformations in various types of cancers [28–30]. Therefore, we obtained integral methylation level of LINE-1 promoters in the samples under study to see whether expression level of PIWI genes impacts their function. Indeed, methylation level of LINE-1 correlates with expression of PIWI genes: promoters of these retrotransposons are hypermethylated in premalignant testis tissues adjacent to nonseminomas unlike those adjacent to seminomas (Figure 3). Importantly, since DNA methylation is a stable biomarker, it could be used as a novel and robust diagnostic tool for distinguishing between seminomas and nonseminomas, possibly, noninvasively (e.g., in seminal fluid). Importantly, this finding could also imply the fact that loss of LINE-1 silencing is a contributor into seminoma but not nonseminoma development.

### More complex role of DNA methylation in regulation of PIWIL1 and PIWIL2 expression

**Figure 4:**
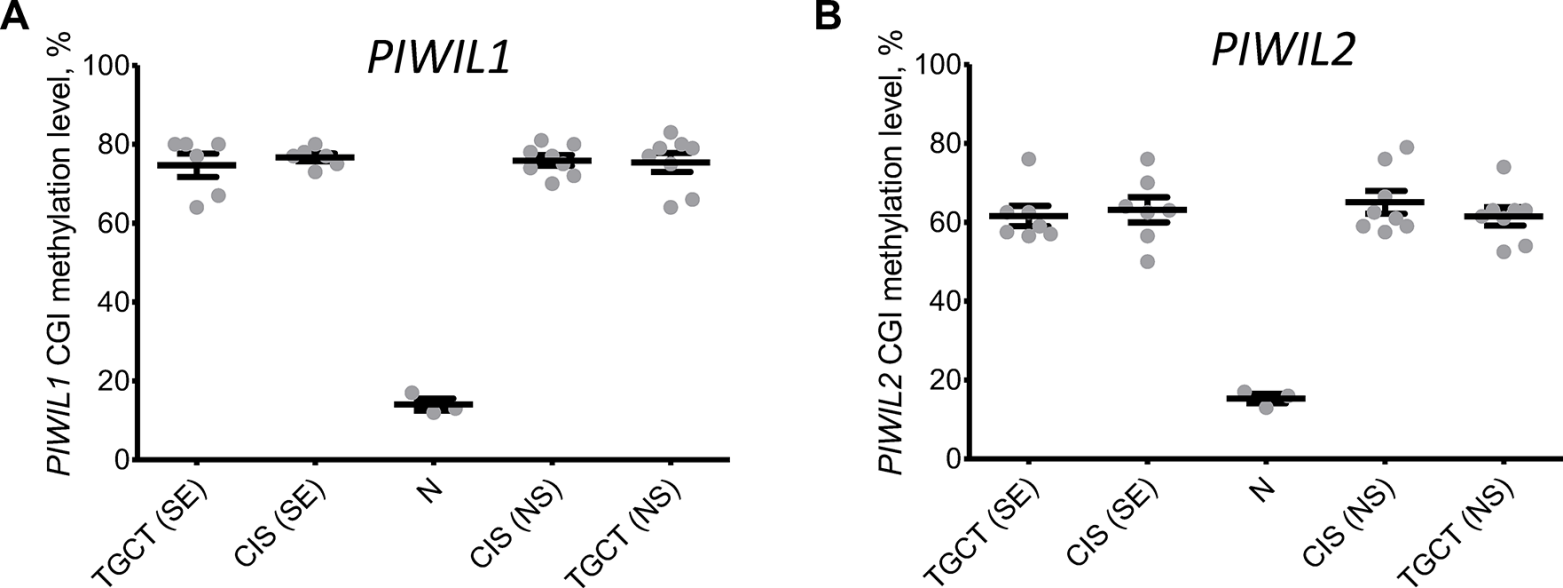
Integral methylation status of *PIWIL1* and *PIWIL2* CpG islands Melting curve analysis was used to obtain methylation level of *PIWIL1* (**A**) and *PIWIL2* (**B**) CpG islands in healthy testis tissues (N samples), premalignant testis tissues (CIS samples) and testicular germ cell tumors (TGCT). SE – seminoma, NS – nonseminoma.

Ferreira *et al.* [12] also examined CGI methylation of PIWI genes promoters and reported an association between CGI methylation and transcriptional repression of the corresponding genes in TGCT samples. Since we could expand these data by examining CIS samples (preneoplastic testis tissue adjacent to tumors), we assessed *PIWIL1* and *PIWIL2* CGI methylation status in our cohort. The results obtained for healthy testis tissues (N samples) and TGCTs were in line with those by Ferreira *et al.* (Figure 4A and 4B) [12]. However, premalignant testis samples adjacent to both seminoma and nonseminoma were highly methylated (Figure 4A and 4B), despite the fact that *PIWIL1* and *PIWIL2* are expressed in CIS samples adjacent to nonseminomas at the level comparable with healthy testis tissues (N samples). This disagreement could be caused by a more complex link between *PIWIL1* and *PIWIL2* CGI methylation and gene expression.

**Figure 5:**
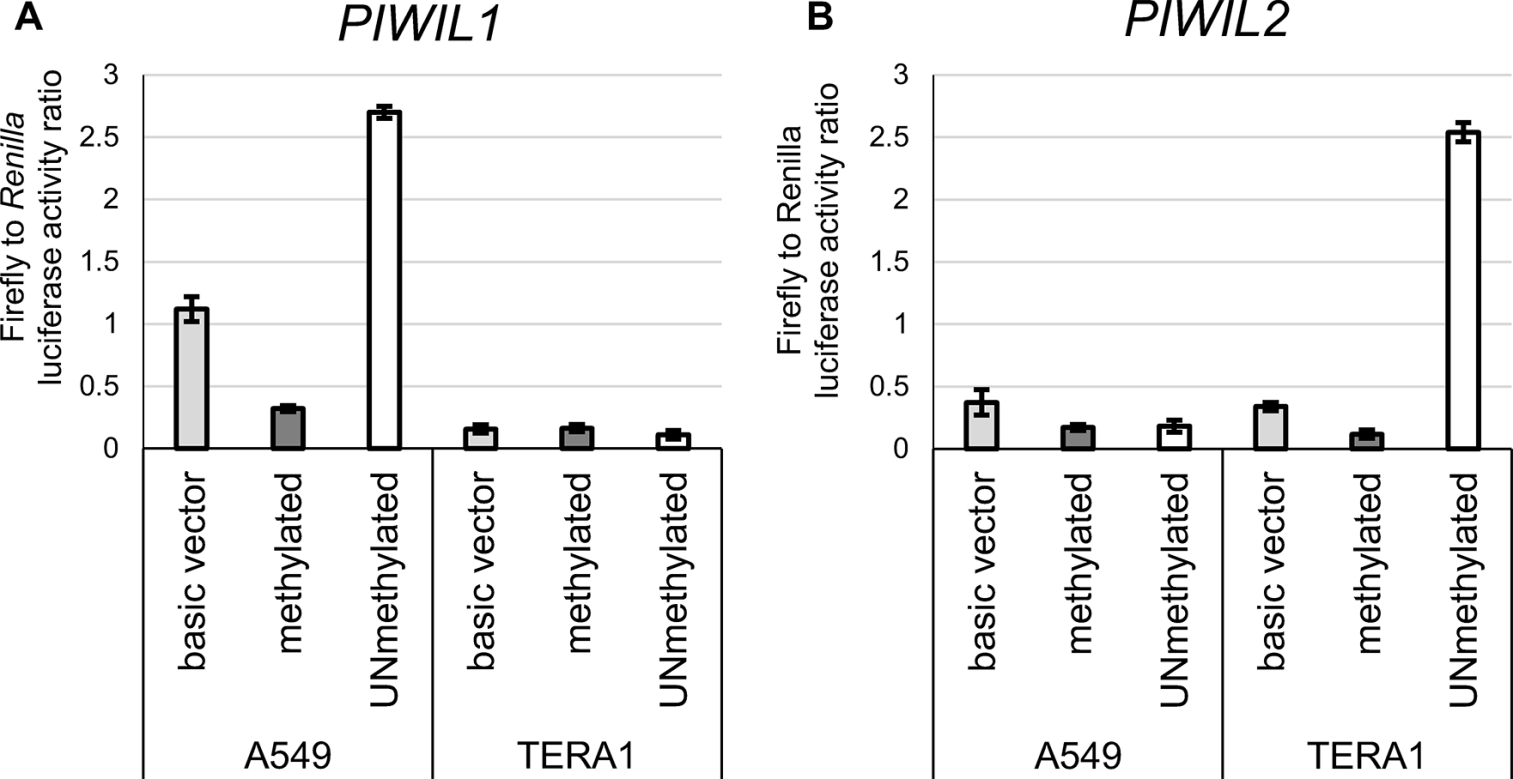
Luciferase reporter assay with methylated or unmethylated *PIWIL1* and *PIWIL2* promoters Luciferase reporter constructs pGL4.10 with either methylated or unmethylated PIWIL1 (**A**) or PIWIL2 (**B**) promoter were transfected into TERA1 and A549 cells. The basic vector (no promoter control) was used as the reference.

To test for this hypothesis, we performed two experiments *in vitro* in cancer cell lines. In the first assay, lung carcinoma A549 cells (TGCT-irrelevant control) and embryonal carcinoma TERA1 cells (nonseminoma-like cells) were transiently transfected with a luciferase reporter construct containing unmethylated or methylated *PIWIL1/2* promoters designed according to Dell *et al.* [31]. Importantly, these constructs were exogenous for cells and devoid of chromatin “memory”. As expected, the methylated promoters exerted no activity; however, the unmethylated *PIWIL1* promoter was only active in A549 cells and unmethylated *PIWIL2* promoter was only active in TERA1 cells (Figures 5A and 4B), suggesting that CGI unmethylated status is necessary but not sufficient for *PIWIL1/2* promoter activity, possibly, because of lack of transcriptional factors essential for transcriptional activation.

**Figure 6:**
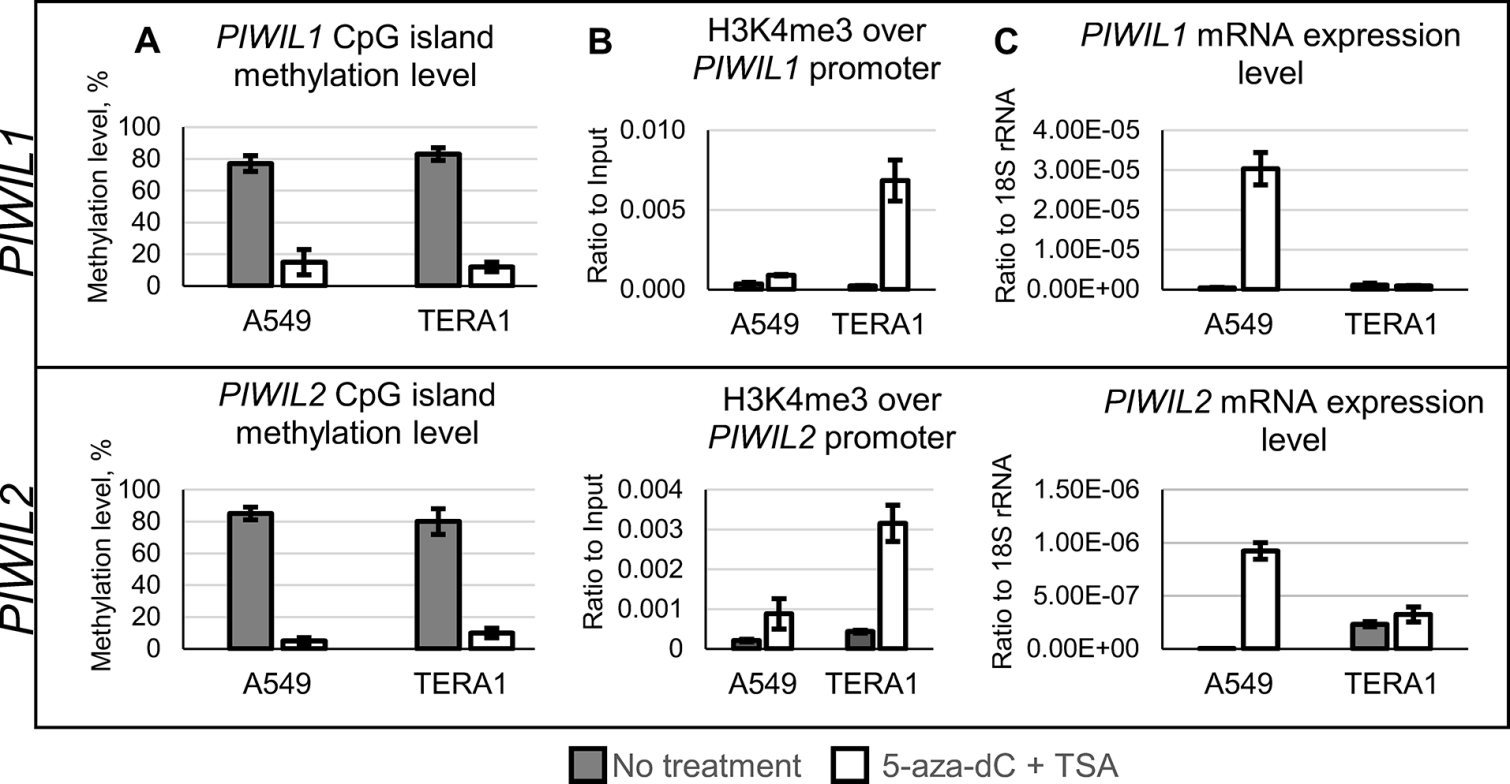
Drug-induced demethylation of genomic promoters of *PIWIL1* and *PIWIL2* TERA1 and A549 cells were treated with 5-Aza-2′-deoxycytidine (5-aza-dC) and trichostatin A (TSA) and analyzed for promoter CpG methylation (**A**), chromatin modifications of active transcription start sites (H3K4me3, trimethylated lysine 4 in histone 3) along promoters (**B**), and transcription level (**C**) of *PIWIL1* and *PIWIL2* genes.

In the second experiment, we wanted to assess how promoter DNA methylation influences expression of *PIWIL1* and *PIWIL2* in their natural genomic DNA context. While studying *PIWIL1* and *PIWIL2* promoters in the genome of TERA1 and A549 cell lines, we found them to be highly methylated. In order to achieve their demethylation, these cell lines were treated with inhibitors of DNA methyltransferase (5-Aza-2′-deoxycytidine) and histone deacetylases (trichostatin A) [32]. The combined drug treatment protocol was used as the most efficient method to ensure almost complete loss of methylated CpGs from around methylated promoters of *PIWIL1/2* (Figure 6A).

As expected, in A549 cells promoter demethylation led to an increase in H3K4me3 (histone methylation mark of active transcription, Figure 6B) and upregulated *PIWIL1* and *PIWIL2* expression (Figure 6C).

However, in TERA1 cells *PIWIL2* transcription was already active before drug treatment and in presence of its highly methylated promoter (Figure 6A and 6C). Drug-induced demethylation did not produce any changes in transcription for both *PIWIL1* and *PIWIL2* (Figure 6C). That is a surprising result because H3K4me3 chromatin marks for active transcription were gained on both promoters (Figure 6B). Among plausible explanations for the results in TERA1 cell line may be presence of other than CpG methylation mechanisms that maintain activating or repressive chromatin environment on *PIWIL1/2* promoters.

Overall, these seemingly conflicting findings in *in vitro* experiments imply a more complex link between CGI methylation in the promoter regions and *PIWIL1/2* transcription and, possibly, interplay with other expression regulatory systems. That is not an entirely new observation, since “atypical” promoters (active, while being heavily methylated) have been described in the course of spermatogenesis [33]. Moreover, active transcription from promoters associated with methylated CGIs was also shown in other contexts [34–38].

## CONCLUSION

In this work, we extended results from previous studies on PIWI machinery proteins expression in TGCTs by introducing an intermediate premalignant stage. Namely, we found that *PIWIL1/2/4* and *DDX4* are concertedly expressed in preneoplastic testis tissue adjacent to nonseminoma but are downregulated in those adjacent to seminoma. Possibly, the loss of spermatogenesis and/or PIWI machinery might be required for seminoma development. This attrition could also take place at an earlier stage of neoplastic transformation into seminoma, unlike nonseminoma. Importantly, whether it is a cause or a consequence remains to be answered in further studies. In addition, TGCTs accompanied by cryptorchidism without orchiopexy are most commonly seminomas [39–41]. Therefore, reasons for impaired spermatogenesis in uncorrected cryptorchidism and development of seminoma might be similar and connected with piRNA pathway function.

Furthermore, PIWI proteins expression pattern in premalignant testis was found to have an impact on piRNA machinery function - methylation level of LINE-1 promoters, which could directly impact the genomic stability and, in turn, promote malignization of germ cells. Apart from that, this observation can be used as a novel diagnostic biomarker for the two types of TGCTs.

Moreover, use of matched CIS/TGCT pairs also allowed minimizing data variability and obtaining a refined picture devoid of patient-to-patient heterogeneity. Finally, a more complex role of *PIWIL1/2* CGI methylation in expression of corresponding genes was revealed.

## MATERIALS AND METHODS

### Sample collection

Twenty two pairs of TGCT tissues and corresponding adjacent normal testicular parenchyma were obtained from orchiectomy specimens: 7 seminomas (patient age ranges between 19 y.o. and 51 y.o. with a median of 34 y.o.) and 15 nonseminomas (patient age ranges between 18 y.o. and 49 y.o. with a median of 28 y.o.). The 15 nonseminomas included 4 teratomas, 1 choriocarcinoma, 1 embryonal carcinoma and 9 TGCTs with mixed histology. 5 samples of normal testis tissue were obtained from prostate cancer patients undergoing surgical castration (patient age ranges between 53 y.o. and 84 y.o. with a median of 68 y.o.). The samples were immediately frozen in liquid nitrogen. All patients provided written informed consent according to the federal law, and the study was approved by the ethical committees of the Shemyakin-Ovchinnikov Institute of Bioorganic Chemistry of the Russian Academy of Sciences and Blokhin Russian Cancer Research Center after reviewing patients' consent and information forms.

### Gene expression analysis by qRT-PCR

Total RNA extraction and purification was performed with Trizol reagent (Invitrogen, USA). cDNA synthesis was performed with MintReverse Transcriptase and following qPCRs with qPCRmix-HS SYBR system (Evrogen, Russia) on Lightcycler 480 (Roche, USA) according to the manufacturers' instructions. Primer pairs used in amplification are listed in Supplementary Data File 4. The assays were performed in triplicates.

### DNA methylation analysis by melting curve assay

Melting curve analysis was used to assess the integral level of DNA methylation for LINE-1 and *PIWIL1/2* promoter CGIs as previously described [42]. Primers are listed in Supplementary Data File 5 and calibration curves are shown in Supplementary Data File 6. Biological duplicates (cell line experiments only) and technical triplicates were used to ensure reproducibility.

### Luciferase reporter assay

*PIWIL1/2* promoter regions were cloned (primers are listed in Supplementary Data File 7) and ligated upstream of the luciferase reporter gene in pGL4.10 (Promega, USA) in either methylated or unmethylated state (according to Dell *et al.* [31]). Transient cotransfection with pRL-TK reference vector (Promega, USA) coding for *Renilla reniformis* luciferase was performed using Lipofectamin 2000 (Invitrogen, USA) according to the manufacturers' protocols. Relative luciferase activity was obtained. Biological and technical duplicates were used to ensure reproducibility.

### Cell lines and treatment with 5-Aza-dC and trichostatin A

Cell lines TERA1 (ATCC HTB-105) and A549 (ATCC CCL-185) were purchased from ATTC (USA) and maintained in DMEM/F12 (1:1) (Invitrogen, USA) supplemented with 10% FCS (Invitrogen). For DNA demethylation experiments, cells were grown for 4 days in the presence of freshly prepared 5 μM 5-Aza-2′-deoxycytidine (Sigma, USA); the medium containing 5-Aza-dC was changed daily. Trichostatin A (Sigma, USA) was added at the concentration of 200 nM 8 h before analysis. Untreated cell cultures were used as controls. Biological and technical triplicates were used to ensure reproducibility.

### Chromatin immunoprecipitation (ChIP) assay

ChIP was performed as described earlier [43] using antibodies to trimethylated lysine 4 in human histone 3 (H3K4me3; ab8580, Abcam, USA). Biological and technical triplicates were used to ensure reproducibility. Primer pairs are listed in Supplementary Data File 8.

## SUPPLEMENTARY MATERIALS DATA FILES



## Abbreviations

TGCT: testicular germ cell tumor
ChIP: chromatin immunoprecipitation
H3K4me3: histone 3 lysine 4 trimethylation.
