# Supplementary material for: Distinguishing epigenetic features of preneoplastic testis tissues adjacent to seminomas and nonseminomas
Source: Oncotarget. 2016 Jan 29;7(16):22439–47. doi: 10.18632/oncotarget.7074 (PMC5008371; doi:10.18632/oncotarget.7074)
Supplement: Supplementary file 1 [file oncotarget-07-22439-s001.pdf]

Distinguishing epigenetic features of preneoplastic testis tissues adjacent to seminomas and nonseminomas

Supplementary Materials

Supplementary Data File 1: Spearman's correlation coefficient for the expression of PIWI pathway genes (*PIWIL1*, *PIWIL2*, and *PIWIL4*), germ cell marker *DDX4* (homologue of VASA), and CIS (carcinoma *in situ*) markers *POU5F1* (OCT3/4) and *NANOG* in healthy testis tissues and histologically normal testis tissues adjacent to TGCTs

	PIWIL1	PIWIL2	PIWIL4	DDX4	POU5F1	NANOG
PIWIL1						
PIWIL2	0.98					
PIWIL4	0.86	0.85				
DDX4	0.99	0.97	0.85			
POU5F1	−0.40	−0.45	−0.54	−0.40		
NANOG	−0.20	−0.22	−0.29	−0.20	0.72	

Supplementary Data File 2: Spearman's correlation coefficient for the expression of PIWI pathway genes (*PIWIL1*, *PIWIL2*, and *PIWIL4*), embryonal carcinoma (nonseminoma) marker *SOX2* and seminoma marker *SOX17* in testicular germ cell tumors

	PIWIL1	PIWIL2	PIWIL4	SOX2	SOX17
PIWIL1					
PIWIL2	0.51				
PIWIL4	−0.02	−0.47			
SOX2	−0.47	−0.09	0.05		
SOX17	0.32	0.64	−0.39	−0.41	

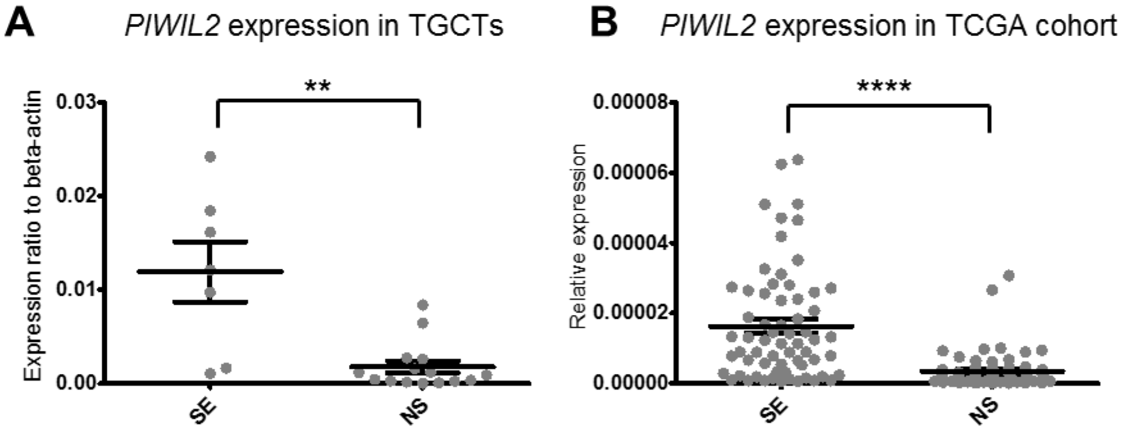

Supplementary Data File 3: Expression of *PIWIL2* in testicular germ cell tumors (SE – seminomas, NS – nonseminomas). (**A**) Relative to β-actin expression of *PIWIL2* in testicular germ cell tumor samples used in the study ($n = 22$). (**B**) Relative expression of *PIWIL2* in The Cancer Genome Atlas (TCGA) cohort ($n = 127$). P value summary for Mann-Whitney non-paired U test is shown.

Supplementary Data File 4: Primer pairs used in qRT-PCR

Primer pair name	Sequences	Product size, bp
PIWIL1	GCAAAAGGTCACAGCAGACA and CCTCCCATCTTGCAGTTCAT	204
PIWIL2	CAGGCAGAGGCCATGTATTT and CTCGGAACATGGAGACCAAA	96
PIWIL4	ATGCTCACAAGCTGACCTT and TTCCTAGACATGAAAACTAAGC	213
POU5F (OCT3/4)	TGCAGCAGATCAGCCACAT and TAGTCGCTGCTTGATCGCTT	106
NANOG	GCTGAAGAATAGCAATGGTG and AGTCGGGTTCACCAGGCA	97
DDX4	AAAGTGGATTTGCCTCTGGG and CTCCAAAACCACCCATTGTG	93
SOX2	ATGCGGGATACGCCAGTGAC and GCTCTGCCTCCTCCACGAAG	354
SOX17	GATGCACAACTCGGAGATCAG and GGCAGCGTGTACTTATCCTTCT	189
ACTB(beta-actin)	GAGCGGGAAATCGTGCGTGACATT and GATGGAGTTGAAGGTAGTTTCGTG	234

Supplementary Data File 5: Primer pairs used to assess *PIWIL1*, *PIWIL2* and LINE-1 CpG island methylation status (melting curve analysis)

Primer pair name	Sequences	Product size, bp
PIWIL1CGI	CGGGGACCTGAGAGCTCT and CGCCTCACTTGGGGTCCT	188
PIWIL1CGI (bisulfite)	CGGGGATTTGAGAGTTTT and CGCCTCACTTAAAATCCT	
PIWIL2CGI	GAAATTGGCCCTGGAGCA and CAACCCTCAGCAGCTCCC	243
PIWIL2CGI (bisulfite)	CGGAAATTGGCCCTGGAGT and CGCAACCCTCAACAACTCCC	
LINE-1 CGI	GCAAGGGGTCAGGGAGTTCCCTTT and AGGACCCTCTGAGCCAGGTGTGGGATATA	156
LINE-1 CGI (bisulfite)	GTAAGGGGTTAGGGAGTTTTTTTT and AAAACCCTCTAAACCAAATATAAAATATA	

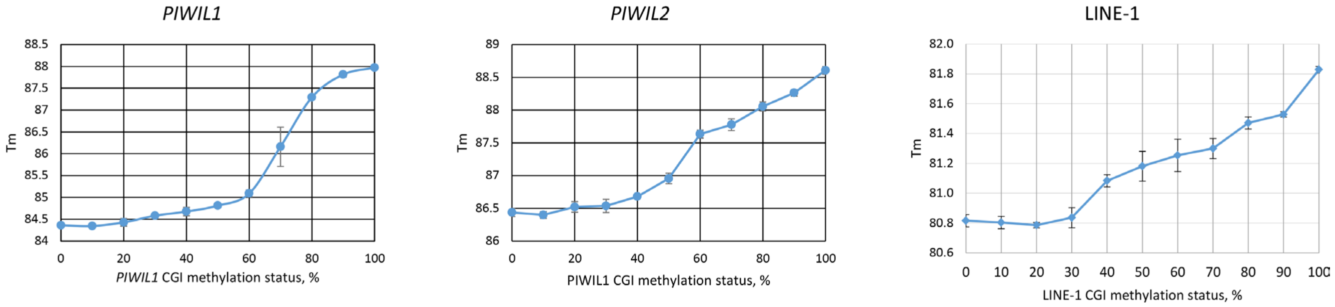

Supplementary Data File 6: DNA methylation analysis of *PIWIL1*, *PIWIL2* and LINE-1 CpG islands by melting curve assay (calibration curves)

Supplementary Data File 7: Primer pairs used for cloning *PIWIL1* and *PIWIL2* promoter regions

Primer pair name	Sequences	Product size, bp
*PIWIL1*promoter	GTGGGGGCGGGGCTCCTC and TCAGCCTGGCCCCGCAC	372
*PIWIL2*promoter	GGGCAAGTCAATTTCAACACGC and CACAGCCCTGCCAGGGGT	665

Supplementary Data File 8: Primer pairs used for qPCR in the H3K4me3 ChIP assay

Primer pair name	Sequences	Product size, bp
*PIWIL1*promoterChIP	CGTTTTTAAGTTGTGGCCA and CGCTACCAGCTCTGCCAA	161
*PIWIL2*promoterChIP	TCACATAACTCCCAGCGCT and GCAAGCTATCTAATTGGCCCAT	102
